# Silicon-graphene conductive photodetector with ultra-high responsivity

**DOI:** 10.1038/srep40904

**Published:** 2017-01-20

**Authors:** Jingjing Liu, Yanlong Yin, Longhai Yu, Yaocheng Shi, Di Liang, Daoxin Dai

**Affiliations:** 1 Centre for Optical and Electromagnetic Research, State Key Laboratory for Modern Optical Instrumentation, Zhejiang Provincial Key Laboratory for Sensing Technologies, Zhejiang University, Zijingang Campus, Hangzhou, China; 2 Shenzhen Kuang-Chi Institute of Advanced Technology, Shenzhen, China; 3 Shenzhen Key Laboratory of Transformation Optics and Spatial Modulation, Shenzhen, China; 4 Hewlett Packard Labs, Hewlett Packard Enterprise, Palo Alto, CA, USA

## Abstract

Graphene is attractive for realizing optoelectronic devices, including photodetectors because of the unique advantages. It can easily co-work with other semiconductors to form a Schottky junction, in which the photo-carrier generated by light absorption in the semiconductor might be transported to the graphene layer efficiently by the build-in field. It changes the graphene conduction greatly and provides the possibility of realizing a graphene-based conductive-mode photodetector. Here we design and demonstrate a silicon-graphene conductive photodetector with improved responsivity and response speed. An electrical-circuit model is established and the graphene-sheet pattern is designed optimally for maximizing the responsivity. The fabricated silicon-graphene conductive photodetector shows a responsivity of up to ~10^5 ^A/W at room temperature (27 °C) and the response time is as short as ~30 μs. The temperature dependence of the silicon-graphene conductive photodetector is studied for the first time. It is shown that the silicon-graphene conductive photodetector has ultra-high responsivity when operating at low temperature, which provides the possibility to detect extremely weak optical power. For example, the device can detect an input optical power as low as 6.2 pW with the responsivity as high as 2.4 × 10^7^ A/W when operating at −25 °C in our experiment.

Graphene is a two-dimensional mono-layer material and has attracted intensive attention owing to its unique optoelectronic properties^1^,^2^,^3^,^4^, such as high carrier mobility (~200,000 cm^2^V^−1^s^−1^ at room temperature)^5^,^6^, zero-bandgap and electrochemically tunable Fermi level^4^,^7^, broadband absorption of π*α* = ~2.3% per layer for normal incidence illumination^3^,^4^, and high optical nonlinearities^8^,^9^. Furthermore, graphene can co-work with some conventional semiconductors to form a Schottky junction^10^,^11^,^12^. When light illuminates the semiconductor/graphene Schottky junction^13^, the photo-carriers can transport from the conductor layer to the graphene layer by the build-in field. As a result, the graphene conduction changes according to equation, Δ*σ* = Δ*neμ*^14^, where Δ*n* is the carrier-*concentration* variation in graphene, *μ* is the carrier mobility, *e* is the electron charge. This provides a platform to realize a graphene-based conductive-mode photodetector with high responsivity. For example, Konstantatos *et al*.^15^ realized a hybrid graphene conductive photodetector with very high responsivity by combining the high absorption of quantum-dots and the high mobility of graphene. For this quantum-dots/graphene photodetector, the responsivity is up to ~5 × 10^7 ^A/W for an input optical power less than 10 fW, while the response is slow (in the order of ~10 ms) due to carrier trapping effect of quantum dots. Recently, a silicon-based graphene conductive photodetector was demonstrated by putting a graphene sheet on an N-type silicon substrate with a doping level *n* < 10^16 ^cm^−3^ (the resistivity *ρ* ≥ 10 Ω·cm). For this photodetector, the response time is ~1.48 ms, while the responsivity is reduced to be ~10^6^ A/W under an input optical power less than 10 pW^16^. Similarly, Chen *et al*. demonstrated a silicon-graphene conductive photodetector with further improved response time around 3 μs by using a lightly-doped P-type silicon substrate while the responsivity further decreases to ~10^4^ A/W under an input optical power of 0.112 μW^17^.

In this paper, we design and demonstrate an improved silicon-graphene conductive photodetector with high responsivity as well as response speed. Here we choose a silicon substrate with a relatively high N-type doping level of ~7 × 10^15 ^cm^−3^ (the resistivity *ρ* is 0.55 ~ 0.8 Ω·cm), and the graphene pattern is designed specially to have optimized metal/graphene contact resistance according to an established electrical-circuit model. The fabricated silicon-graphene conductive photodetector with an optimized graphene-sheet pattern shows a responsivity up to10^5 ^A/W for an input optical power P = 10 nW at room temperature (27 °C) and the response time as short as 30 μs. The temperature dependence of silicon-graphene conductive photodetectors is also studied for the first time. It is found that the responsivity increases greatly as the temperature decreases. Our experimental result shows that the silicon-graphene conductive photodetector has ultra-high responsivity when operating at relatively low temperature, and thus enables the detection of ultra-low optical power. For example, when operating at −25 °C, the device can detect an input optical power as low as P = ~ 6.2 pW and the corresponding responsivity is as high as 2.4 × 10^7^ A/W.

## Structure and Result

**Figure 1:**
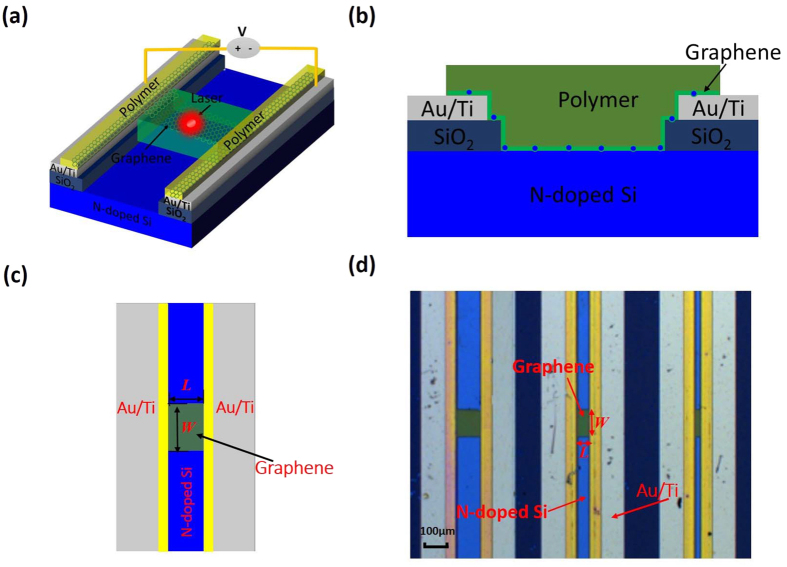
(**a**) Three-dimensional schematic of the present silicon-graphene conductive photodetector with silicon oxide between silicon and electrodes, graphene on the electrode. (**b**) The cross section of the photodetector showing the regions with silicon, silicon dioxide, electrodes, graphene and protection polymer. (**c**) The schematic top view of the photodetector. *L* and *W* are the length and the width of the graphene sheet on silicon, respectively. (**d**) The microscope image of the fabricated silicon-graphene conductive photodetector.

Figure 1(a) shows the three-dimensional schematic configuration of the silicon-graphene conductive photodetector, which is with a silicon substrate with a relatively high N-type doping level of ~7 × 10^15 ^cm^−3^. Figure 1(b) shows the schematic cross section of the photodetector. There is a 100 nm-thick SiO_2_ layer formed on the silicon substrate by utilizing a thermal oxidation process. This SiO_2_ layer is removed selectively to open a window so that the graphene sheet can contact with the silicon substrate directly. The Ti/Au electrodes were fabricated with a sputtering process. The thicknesses of the Ti and Au layers are 5 nm and 80 nm, respectively. A monolayer of graphene sheet grown by chemical vapor deposition (CVD) was then wet-transferred onto the top of the chip and was patterned by an oxygen plasma etching process. The graphene-sheet pattern of the photodetector is shown in Fig. 1(c) (top view). The graphene transferred to the silicon substrate is in a good condition, which can be examined by using the popular Raman spectroscopy technique. Here, the length *L* and the width *W* of the graphene sheet on silicon are chosen as 100, 50, 20, and 10 μm, respectively. Finally the negative polymer layer used for patterning the graphene layer is kept for graphene protection. A top-view microscopic image of the fabricated device is shown in Fig. 1(d).

**Figure 2:**
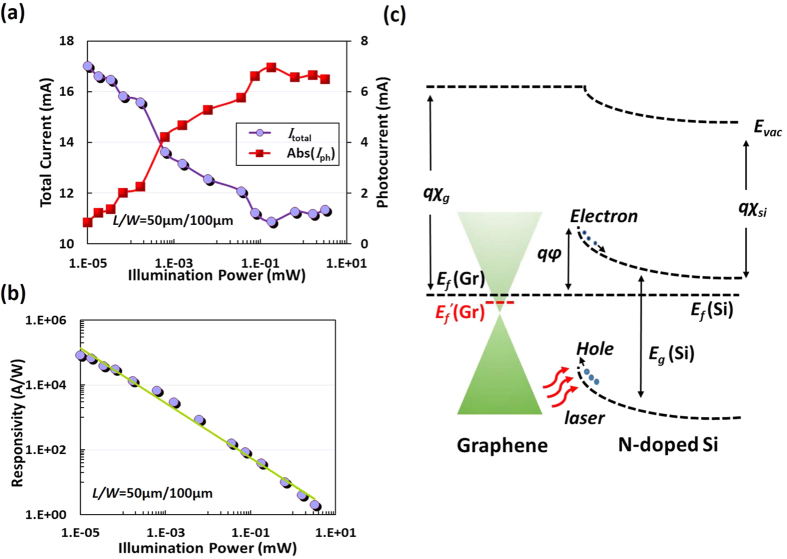
(**a**) The measured total current *I*_tot_ (see the circles ○), the photocurrent |*I*_ph_| (see the squares □); (**b**) The measured responsivity with different optical power when operating at a bias voltage of 5 V; (**c**) The band diagram of the Graphene/N-doped Silicon junction.

For the devices characterization, a red-light source (whose wavelength is 635 nm) with a fiber pigtail was used as the light source to illuminate the chip from the top side. The output power of the laser ranges from −60 dBm to 16 dBm and the illuminating spot size to the graphene surface is similar to the diameter of the fiber mode spot-size, which is smaller than 20 μm. Figure 2(a) shows the measured total current *I*_total_ of the silicon-graphene photodetectors with *L*/*W* = 50 μm/100 μm under a bias voltage of V_bias_ = 5 V as a function of the laser power varying from 10 nW to 3.2 mW. Here the operation temperature is fixed as 27 °C and the measured dark current is *I*_dark_ = ~17.8 mA (without light illumination, i.e., P = 0 mW). The measured responsivity as high as 0.83 × 10^5 ^A/W when P = 10 nW. As shown in Fig. 2(a), when the input optical power increases, the total current *I*_total_ decreases, which is different from the results of graphene/P-doped silicon photodetector reported previously^17^. For graphene/P-doped silicon photodetector demonstrated in ref. 17, the total current *I*_total_ rises with increasing input optical power and is larger than the dark current *I*_dark_. This can be explained from the carrier transport in the Schottky junction formed between the graphene sheet and the silicon substrate. In our design, when the graphene sheet contacts with the N-doped silicon substrate, electrons transport from the silicon substrate to the graphene sheet according to the schematic energy-band diagram (in Fig. 2(c)), resulting in the Fermi-level shift of the graphene sheet. The Fermi-level shift can be large enough to make the graphene sheet become N-type doping since the silicon substrate has a relatively large N-type doped level of ~7 × 10^15^ cm^−3^. When there is laser illumination, electron-hole pairs are generated in the depletion region in silicon and separated by the build-in field. Finally, the holes transport from the silicon substrate to the graphene sheet, as shown in Fig. 2(c). The recombination of these holes with the electrons in the graphene happens and thus the carrier concentration in the graphene sheet decreases. Based on a well-known Eq. (1) below, the resistance *R*_G_ of a graphene sheet can be calculated as


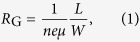


where *n* is the carrier concentration in graphene. It can be seen that the graphene resistance increases as the carrier concentration decreases. This is why we observed that the total current *I*_total_ with light illumination becomes less than the dark current *I*_dark_. As the optical power increases further to a certain value P_0_ (e.g., here P_0_ = ~0.18 mW as shown in Fig. 2(a)), the Fermi-level of graphene approaches the Dirac point and the graphene resistance reaches its maximum, leading to a minimal total current *I*_total_ around 10.9 mA. When further increasing the optical power beyond 0.18 mW, more holes transport from the silicon substrate to the graphene sheet. The graphene Fermi-level further reaches a point below the Dirac point, so that the graphene sheet becomes P-type doping. In this case, the resistance decreases as the optical power increases and consequently the total current increases, as shown in Fig. 2(a). We also observed the total current becomes saturated when the optical power is higher than 0.64 mW. This is because the barrier height in the silicon-graphene Schottky diode becomes disappeared, which happens when the Fermi-level shift of graphene is large enough due to the high optical power illumination.

**Figure 3:**
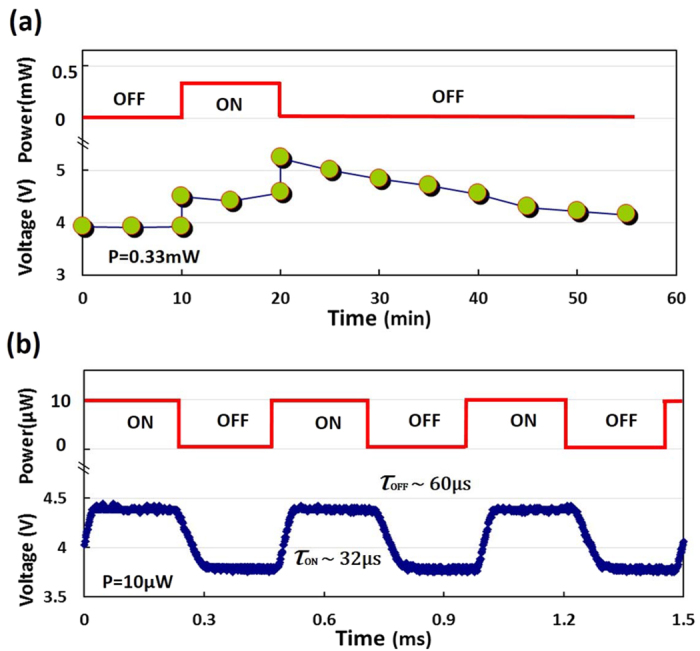
The measured temporal voltage response for the photodetector under (**a**) high optical power illumination (P = 0.33 mW), and (**b**) low optical power illumination (P = 10 μW). Here ON (OFF) means that light illumination is on (off).

We also measure the dynamic response of the silicon-graphene photodetector (see the details for the measurement in the *Method* part below). For the measurement, we use a current source to fix the photodetector bias current I_bias_ at 10 mA and an oscilloscope to monitor the temporal dynamics of the device voltage under different illumination situations. We consider two cases with different optical powers P_1_ and P_2_, where P_1_ < P_0_ and P_2_ > P_0_. As discussed above, here P_0_ is the optical power to induce the Fermi-level of graphene to approach the Dirac point (i.e., the graphene resistance becomes maximal. For the present case, one has P_0_ = 0.18 mW (see Fig. 2(a)). As a result, we choose P_1_ = 10 μW, and P_2_ = 0.33 mW for the measurement. Figure 3(a) and (b) show the measured dynamic responses under 0.33 mW and 10 μW illumination, respectively. For the case of P_2_ = 0.33 mW (P_2_ > P_0_), the measured voltage increases quickly when light illumination is switched from *off* to *on*. However, when light illumination is switched from *on* to *off*, the measured voltage show a sudden rise and then decreases very slowly, which is mainly due to the majority carrier trapping effect at high laser power^18^. Initially, the graphene sheet contacting with the silicon substrate becomes N-type doped in the dark case. When the light illumination is *on* with an optical power P_2_ > P_0_ (e.g., P_2_ = 0.33 mW here), the graphene sheet becomes P-doped because of the carrier transport through the silicon-graphene Schottky junction, as explained above (see Fig. 2(a)). When the light illumination is switched *off* suddenly, this P-doped graphene is likely to return back to the initial state with N-doping through the hole-electron combination process. Therefore, the resistance of the graphene sheet climbs to a maximal value (i.e., when the Fermi-level is at the Dirac point) and then decreases. Correspondingly, the device voltage increases first and then decreases temporally, as observed in our experiments. Note that the electron-hole combination process lasts for a very long time due to the electron trapped in the N-doped silicon region^18^. We observe that it takes more than 35 minutes for the voltage to be recovered to the original level measured in the dark case. Therefore, it has been limited when used for high-speed photodetection under a relatively high optical power. For the case of P_1_ = 10 μW (P_1_ < P_0_) in Fig. 3(b), when the light illumination is *on (off*), the voltage becomes high (low) as expected. The rising time τ_ON_ and the decay time τ_OFF_ are about 32 μs and 60 μs, respectively. The response time is mainly determined by the processes of the carrier transport through the silicon-graphene junction and in graphene. It can be seen that this response is much faster than that of quantum-dots/graphene photodetector (~10 ms)^15^.

One should note that the photodetector’s responsivity strongly depends on the geometrical structure parameters. In order to understand how the responsivity depends on the geometrical structure parameters of the photodetector, an electrical-circuit model is developed below. One has


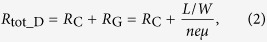



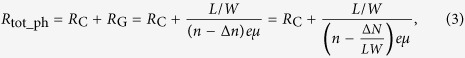



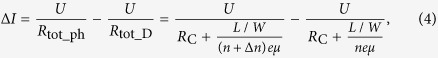


where *R*_tot_D_ (*R*_tot_ph_) is the total serial resistance of the photodetector without (with) light illumination, *R*_C_ is the contact resistance of the graphene sheet with the Au/Ti electrode, *n* is the carrier concentration in graphene, Δ*N* is the carrier *number* injected to graphene from silicon, Δ*n* is the carrier-*concentration* variation in graphene, and *I*_ph_ is the photocurrent. When the illumination power is weak, the carrier-*concentration* variation in graphene, Δ*n*, is much lower than the carrier concentration *n* in graphene, Eq. (5) can be simplified as


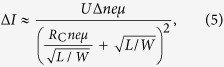


**Figure 4:**
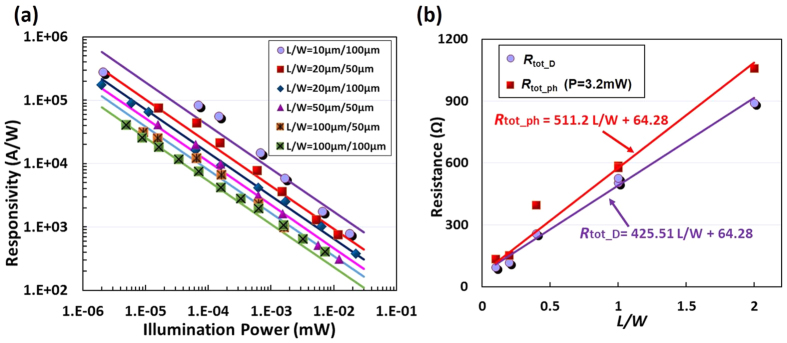
(**a**) The measured responsivity for the photodetectors with different graphene sheet dimensions and aspect ratios (*L*/*W*) as a function of different illumination power; (**b**) The measured total resistance *R*_tot_ph_ with an optical power P = 3.2 mW (see the squares □), and *R*_tot___D_ when the light illumination is on or off (see the circles ○).

According to this simplified equation, if the *L*/*W* is fixed in the design, the current increment *I*_ph_ is proportional to Δ*n*. This indicates that the responsivity can be improved by reducing the graphene area to enhance the carrier-concentration variation Δ*n* in the graphene. Note that the graphene area should not be smaller than the spot size of light illumination in order to receive all optical power. On the other hand, when the graphene area is fixed in the design, the current variation *I*_ph_ varies with the ratio *L*/*W* hyperbolically. It can be seen that the current variation *I*_ph_ reaches maximum when the *R*_C_ = (*L*/*W*)/*neμ* = *R*_G_. In order to verify the dependence of the responsivity on the graphene size, we measured the responsivity of the fabricated photodetectors with different sizes, i.e., *L*/*W* = 10 μm/100 μm, 20 μm/50 μm, 20 μm/100 μm, 50 μm/50 μm, 100 μm/50 μm, and 100 μm/100 μm, as shown in Fig. 4(a). For example, we consider the two photodetectors with *L*/*W* = 50 μm/50 μm and 100 μm/100 μm, which have different graphene areas but the same ratio *L*/*W*. Therefore, the responsivity for the photodetector with *L*/*W* = 50 μm/50 μm (pink line) is higher because of the smaller area, as shown in Fig. 4(a). Figure 4(b) shows the measured total device serial resistances (*R*_tot_ph_ and *R*_tot_D_) of the photodetectors with different sizes of the graphene sheet when the light illumination is on or off. Here *R*_tot_ph_ and *R*_tot_D_ are the resistances for the cases with the illumination power *P*_in_ = 3.2 mW and 0 mW, respectively. From the data shown in Fig. 4(b), one can easily obtain the square resistance R_□_ and the contact resistance *R*_C_ according to Eqs (2, 3). The square resistance R_□_ is given by the slopes of the R~(L/W) curves shown in Fig. 4(b), and the contact resistance is the value to resistance axis at *L*/*W* = 0. For the present case, one has *R*_C_ = 64.28 Ω, R_□_D_ = 1/(*neμ*) = ~425.5 Ω at P_in_ = 0 mW and *R*_□_ph_ = 1/[(*n*–Δ*n)eμ*] = ~511.2 Ω at P_in_ = 3.2 mW. Regarding the devices with the same graphene area but in different aspect ratios *L*/*W, R*_*G*_ are measured to be ~30 Ω and ~192 Ω for photodetectors with *L*/*W* = 10 μm/100 μm and 20 μm/50 μm. As the resistance of the photodetector with *L*/*W* = 10 μm/100 μm is closer to the contact resistance *R*_C_ (~64.28 Ω), the responsivity is higher as predicted from Eq. (5), which is also verified experimentally in Fig 4(a). When keeping the area *A* = *LW* fixed and choosing the ratio *L*/*W* = 12.2 μm/81.6 μm, the optimal value to achieve *R*_G_ = *R*_C_ according to Eq. (5), one expects to have a maximum responsivity of 2.9 × 10^5 ^A/W under 2 nW illumination.

**Figure 5:**
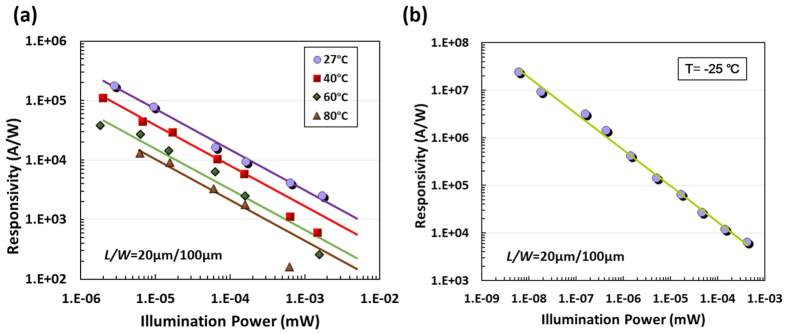
(**a**) The measured responsivity of the photodetectors with *L*/*W* = 20 μm/100 μm when operating at 27 °C, 40 °C, 60 °C, and 80 °C, respectively; (**b**) The measured responsivity of the photodetectors when operating at −25 °C (the highest responsivity is ~2.4 × 10^7^ A/W at 6.2 pW). Here the bias voltage V_bias_ = 5 V.

As the temperature dependence of a photodetector performance is a critical characteristic for practical applications, the silicon-graphene photodetector is also characterized at different temperatures. As an example, Fig. 5(a) shows the measured responsivity of the photodetector with *L*/*W* = 20 μm/100 μm when operating at different temperatures T = 27 °C, 40 °C, 60 °C and 80 °C, respectively. It can be seen that the responsivity decreases notably as the temperature increases. For example, when T = 27 °C, the photocurrent *I*_ph_ is 0.5 mA under an illumination power P = 2.8 nW and the responsivity is 1.78 × 10^5^ A/W. In contrast, when the temperature increases to 80 °C, the photocurrent *I*_ph_ is only 0.07 mA even under a stronger illumination power P = 6.2 nW and the corresponding responsivity is 1.34 × 10^3^ A/W. This temperature dependence of the responsivity is mainly due to the following two factors. First, the metal-graphene contact resistance decreases monotonically as the temperature decreases, due to the high carrier transfer efficiency of nearly ballistic transport at low temperature^19^. For example, the metal (Pt/Au)-graphene contact resistance decreases by ~50% when the temperature decreases from 300 K to 6 K. This improves the photocurrent according to Eq. (5). Second, the graphene resistance decreases linearly with the temperature for graphene suspend^20^ (*n* > 10^11^ cm^−3^) and on the BN substrate^21^, owning to the carrier scattering reduction from acoustic phonons. Therefore, when both the metal contact and graphene resistances increase at high temperature, the device photocurrent drops, which subsequently reduces the responsivity.

Since the responsivity of the photodetector becomes higher when the temperature decreases, here we also characterize the silicon-graphene photodetector when operating at the temperature below 0 °C by setting the thermo electric cooler (TEC). For this measurement, the sample is placed in an enclosed chamber filled with N_2_ gas to prevent moisture condensation or freezing on the chip surface. Figure 5(b) shows the measure responsivity of the photodetector when T = −25 °C. It can be seen that the responsivity increases linearly as the optical power decreases, which is similar to the result when operating at room-temperature as shown in Fig. 2(b). However, in this case, the thermal noise is reduced and very high device sensitivity is achieved. For example, the device can detect an optical power as low as 6.2 pW (−82 dBm) and the corresponding responsivity is 2.4 × 10^7^ A/W. In our experiment, we can not achieve a temperature lower than −30 °C because of the setup limitation. It can be predicted to obtain an even higher responsivity if operating at lower temperature.

## Summary

In summary, we have designed and demonstrated a silicon-graphene conductive photodetector with improved responsivity and response speed. A silicon substrate with relatively high N-type doping level of ~7 × 10^15 ^cm^−3^ has been used and the graphene pattern has been designed optimally to improve the responsivity. An electrical-circuit model has been developed so that one can design the dimension of the graphene-sheet appropriately. The fabricated silicon-graphene conductive photodetector has a response time of as short as 32 μs and a responsivity up to ~10^5 ^A/W for a normal-incident illumination power P = 10 nW at room temperature (27 °C). We have also studied the temperature dependence of the silicon-graphene conductive photodetector for the first time. It is shown that the silicon-graphene conductive photodetector has ultra-high responsivity, which provides the possibility to detect ultra-low optical power when operating at relatively low temperature. The device can detect an optical power as low as P = ~6.2 pW with corresponding responsivity as high as 2.4 × 10^7^ A/W when operating at −25 °C in our experiment. The responsivity for the present silicon-graphene conductive photodetector is expected to be improved further when operating at lower temperature.

## Methods

### Device fabrication

The N-doped Si was oxidized at 1050 °C for one hour to generate ~100 nm-thick SiO_2_ layer. This SiO_2_ layer is removed selectively to open a window by using an electron beam lithography (EBL) process and an wet-etching process with the etchant solution (NH_4_F: HF = 6:1). A second EBL process and a lift-off process were carried out to form the Ti/Au electrodes with the thickness of 5 nm/80 nm. A monolayer of graphene sheet grown by the chemical vapor deposition (CVD) method was then wet-transferred onto the top-surface and patterned by an oxygen plasma etching process.

### Graphene preparation

The monolayer graphene was grown on the copper by the CVD method. A PMMA thin film was formed on the top of the graphene sheet by spin-coating method. Then the copper film was removed by putting it into the Ammonium persulfate solution for 3 hours. After removing the copper film, the graphene-PMMA sample was rinsed on deionized water for ~12 hours, and then dried on the air. Finally, the graphene sheet was wet-transferred and the PMMA film was removed by the acetone.

### Measurement of responsivity and response time

In the experiment, a continuous-wave (CW) 635 nm semiconductor laser with a fiber pigtail was used as the source. The diameter of the illumination spot size is about 20 μm. Keithley 2400 was used to measure the currents (*I*_dark_ and *I*_total_) by tuning the optical power with a variable optical attenuator (VOA). The photocurrent is then given by *I*_ph_ = *I*_total_ − *I*_dark_. In order to measure the photodetector’s response time, an optical chopper was adapted to modulate the CW laser with the frequency of 2 kHz. In the experiment, we measured the voltage by an oscilloscope with a current source (I_bias_ = 10 mA).

## Additional Information

**How to cite this article**: Liu, J. *et al*. Silicon-graphene conductive photodetector with ultra-high responsivity. *Sci. Rep.* **7**, 40904; doi: 10.1038/srep40904 (2017).

**Publisher's note:** Springer Nature remains neutral with regard to jurisdictional claims in published maps and institutional affiliations.
